# Autonomic and circulatory alterations persist despite adequate resuscitation in a 5-day sepsis swine experiment

**DOI:** 10.1038/s41598-022-23516-y

**Published:** 2022-11-11

**Authors:** Marta Carrara, Pietro Antenucci, Shengchen Liu, Andreas Kohler, Rupert Langer, Stephan M. Jakob, Manuela Ferrario

**Affiliations:** 1 Department of Electronics, Information and Bioengineering, Politecnico di Milano, Milan, Italy; 2 Department of Intensive Care Medicine, Bern University Hospital, University of Bern, Bern, Switzerland; 3 Department of Visceral Surgery and Medicine, Inselspital, Bern University Hospital, University of Bern, Bern, Switzerland; 4 Institute of Pathology, University of Bern, Bern, Switzerland; 5 Present Address: Institute of Clinical Pathology and Molecular Pathology, Kepler University Hospital and Johannes Kepler University, Linz, Austria

**Keywords:** Biomarkers, Diseases, Medical research, Engineering

## Abstract

Autonomic and vascular failures are common phenotypes of sepsis, typically characterized by tachycardia despite corrected hypotension/hypovolemia, vasopressor resistance, increased arterial stiffness and decreased peripheral vascular resistance. In a 5-day swine experiment of polymicrobial sepsis we aimed at characterizing arterial properties and autonomic mechanisms responsible for cardiovascular homeostasis regulation, with the final goal to verify whether the resuscitation therapy in agreement with standard guidelines was successful in restoring a physiological condition of hemodynamic profile, cardiovascular interactions and autonomic control. Twenty pigs were randomized to polymicrobial sepsis and protocol-based resuscitation or to prolonged mechanical ventilation and sedation without sepsis. The animals were studied at baseline, after sepsis development, and every 24 h during the 3-days resuscitation period. Beat-to-beat carotid blood pressure (BP), carotid blood flow, and central venous pressure were continuously recorded. The two-element Windkessel model was adopted to study carotid arterial compliance, systemic vascular resistance and characteristic time constant τ. Effective arterial elastance was calculated as a simple estimate of total arterial load. Cardiac baroreflex sensitivity (BRS) and low frequency (LF) spectral power of diastolic BP were computed to assess autonomic activity. Sepsis induced significant vascular and autonomic alterations, manifested as increased arterial stiffness, decreased vascular resistance and τ constant, reduced BRS and LF power, higher arterial afterload and elevated heart rate in septic pigs compared to sham animals. This compromised condition was persistent until the end of the experiment, despite achievement of recommended resuscitation goals by administered vasopressors and fluids. Vascular and autonomic alterations persist 3 days after goal-directed resuscitation in a clinically relevant sepsis model. We hypothesize that the addition of these variables to standard clinical markers may better profile patients’ response to treatment and this could drive a more tailored therapy which could have a potential impact on long-term outcomes.

## Introduction

The autonomic nervous system (ANS) plays a central role in maintaining cardiovascular homeostasis both in physiological and pathological conditions; there is, indeed, a large body of research works reporting a failure of the cardiovascular autonomic control mechanisms during sepsis and septic shock^1–5^. The initial compensatory increase of sympathetic outflow to the heart and peripheral vessels has been shown to become detrimental to the successful correction of hypovolemia and hypotension if prolonged^6–8^. A sustained sympathetic overstimulation is a common phenotype in septic patients, proved also by the high concentration of circulating catecholamines, and it has been found significantly associated to a higher risk of mortality and morbidity^9–12^. This condition of adrenergic stress is not routinely targeted during sepsis resuscitation, although it may be a crucial determinant of many complications secondary from sepsis, such as septic cardiomyopathy, vasopressor resistance, immunomodulation and generally organ dysfunctions^13–15^. In particular, the vascular endothelium regulates the vascular tone, haemostasis and/or vascular permeability, and arterial vascular functions have been reported to be severely impaired during sepsis^16^; recent direct and indirect evidence suggests that sympathetic overactivity and vascular dysfunction may be closely and causally related to each other in a very complex fashion^17^. For example, sympatho-excitatory manoeuvres have been shown to induce endothelial dysfunction and they typically lead to increased stiffness of large arteries^18,19^; on the other side, stiffer arteries may alter the baroreflex function, thus interfering with the sympathetic control of smooth muscle vascular tone^20^. Furthermore, the α1-adrenergic receptors, mainly located in the peripheral vessels, may undergo the desensitization process, a feedback mechanism to protect the receptor against overstimulation, and this further contributes to the failure of autonomic modulation of vascular tone and a less effectiveness of vasopressor therapy^21^. Finally, experimental evidence indicates that the sympathetic ANS is critically influenced, both at central and peripheral level, by the most relevant factors regulating vascular functions, such as nitric oxide (NO), reactive oxygen species (ROS), and endothelin, that are all known to be involved in the dysregulated inflammatory response to sepsis and septic shock^16^.

Previous studies of our group have proposed several indices able to highlight the cardiovascular autonomic dysfunction in different experimental populations during septic shock resuscitation, and we hypothesize that the introduction of these indices to standard therapy targets, such as mean arterial pressure, lactate, and oxygen saturation as suggested by the international SSC guidelines, could be helpful to better evaluate the therapy effectiveness in restoring circulatory characteristics and cardiovascular regulatory mechanisms and to implement an improved resuscitation strategy^22,23^. However, the limited period of observation after the full resuscitation has not permitted to obtain conclusive results, but only an indication about the short-term effects of resuscitation, i.e. about 5/6 h after the resuscitation procedure.

The aim of this work is to characterize the arterial properties and the autonomic cardiovascular response in a long-term swine experiment of polymicrobial sepsis, in order to investigate whether the standard resuscitation therapy was successful in restoring a physiological condition of hemodynamic profile, cardiovascular interactions and autonomic control. Moreover, the analyses exploited the long-time window after resuscitation (72 h) to disentangle and explore possible differences in the early and late response to the therapy.

## Methods

### Animal population, anaesthesia and ventilatory setup

The animal experiment was carried out in collaboration with the Inselspital University Hospital of Bern, Switzerland, and was performed in accordance with the EU Directive 2010/63/EU for animal experiments and the ARRIVE guidelines for animal research, and with the approval of the Animal Care Committee of the Canton of Bern (BE103/16).

Twenty mechanically ventilated and sedated domestic pigs (weight: 39.8 2.7 kg; male/female: 1:1) were used for the experiment. Ketamine (20 mg/kg) and xylazine (2 mg/kg) were intramuscularly administered for animal sedation. General anaesthesia was induced by intravenous infusion of midazolam (0.5–1.0 mg/kg) and atropine (0.02 mg/kg) and maintained with propofol (4–10 mg/kg/h) and fentanyl (3–20 g/kg/h), standard drugs for sedation and analgesia also in critically ill patients. Additional bolus of propofol (20–40 mg) and fentanyl (50–200 g) were administered if necessary, according to sedation depth evaluated hourly by noise pinching and presence of spontaneous movements. After tracheal intubation, the pigs underwent mechanical ventilation in volume-controlled mode, initially with FI_O2_ at 30%, PEEP at 5 cmH_2_O, tidal volume at 8 mL/kg, and respiratory rate at 20 breaths/min and later adjusted with the goal to keep pH between 7.35 and 7.45 and Pa_CO2_ between 35 and 45 mmHg. Gastric output was measured by a gastric probe inserted into the stomach and used also for the later enteral nutrition infusion. During surgical preparation, ringer lactate (RL) solution was infused at 3 mL/kg/h, and measurable blood loss was 1:1 replaced with RL. Rocuronium was administrated (30–50 mg as bolus) only during instrumentation if necessary for better exposure (10 of 20 animals, 5 in sepsis and 5 in sham). After surgeries, 10 mL/kg RL was infused as bolus to all animals to prevent hypovolemia.

### Surgical preparation and data acquisition

The animal was placed in a supine position for instrumentation, a midline neck incision was performed to allow placement of an arterial catheter (5F, Cordis AVANTI, Fremont, CA, USA) in the left carotid artery, a pulmonary artery catheter (8F, Edwards Lifesciences, Irvine, USA) via left external jugular vein, a venous catheter in the left internal jugular vein (5F, Cordis AVANTI, Fremont, CA, USA) for volume infusion, a triple-lumen catheter (7F, Arrow international, Inc, PA, USA) in the right internal jugular vein, and a flow probe (Transonic Systems Inc., Ithaca, NY, USA) around the right carotid artery. Thereafter, a midline laparotomy was performed for placement of a catheter into the urinary bladder for drainage and measurement of urine output. Also, a large-bore drainage tube was placed intraperitoneally for induction of fecal peritonitis.

All pressures were measured and displayed on a multi-modular monitor (S/5 Critical Care Monitor; Datex-Ohmeda, GE Healthcare, Helsinki, Finland). Continuous cardiac output (L/min, by thermodilution) was measured with a Vigilance monitor (Baxter Healthcare Corporation, Edwards Critical Care Division, Irvine, CA), and O_2_ saturations were measured continuously with a Vigilance monitor or intermittently with a blood gas analyzer (Radiometer, Copenhagen, Denmark). Blood flows were measured with ultrasonic transit time perivascular flowmeters using double-channel TS 140 420 flowmeters (Transonic Systems Inc., Ithaca, New York, USA). The signals of all the pressures and blood flows were recorded at 100 Hz by a data acquisition system (Soleasy™; National Instruments Corp., Austin, Tx, USA). More details can be found in^24^.

### Experimental protocol

Instrumentation was followed by a one hour stabilization phase, during which no manipulation on the animal was allowed, thereafter baseline blood samples were taken (time point T1). Subsequently pigs were randomized to fecal peritonitis (SS n = 10)/sham (SH n = 10) and to groups with/without abdominal negative pressure therapy, which was the objective of a separate study with no influence on systemic and splanchnic hemodynamics nor abdominal organ function (all *p* > 0.05, not published). Sepsis was induced by peritoneal instillation of 2 g/kg of autologous feces dissolved in 250 mL warmed glucose 5% solution. After 8 h of observation without resuscitation, another set of blood samples was collected (T2). During stabilization and observation periods, RL infusion rate was reduced to 1.5 mL/kg/h. Before peritonitis induction, to avoid hypovolemia, a bolus of 150 mL of RL was infused to all animals and repeated until there was no fluid response (i.e. increase in cardiac output or stroke volume ≥ 10% after 10 min of fluid bolus). Then, the protocol-based resuscitation was started and continued for approximately 76 h (resuscitation period, RP), including fluid infusion, vasopressor support, electrolyte maintenance, antibiotic therapy, and reduction of body temperature. During the resuscitation period, a basal infusion with RL solution, glucose solution (G50%) and enteral nutrition (36 h after RP) was maintained at 3.0 ml/kg/h. Additional fluid boluses were given to treat hypovolemia. In particular, after 150 ml bolus of RL was given, if cardiac output or stroke volume increased by more than 10%, the fluid response was considered positive, and a repeated fluid bolus was given. This was repeated until the bolus resulted in a negative fluid response. If correction of hypovolemia was not sufficient to keep the mean arterial pressure above 65 mmHg, a continuous infusion of noradrenaline was started to keep the blood pressure at this level, as suggest in the 2013 sepsis guidelines^25^, which were the only available at the time this experimental protocol was designed.

If the mixed venous oxygen saturation (SvO_2_) was ≤ 50%, dobutamine administration was started at a dose of 5 mg/h. This dose was increased by the same amount every 30 min until the SvO_2_ was 50% or higher or until a maximal dose of 20 mg/h was reached. The sepsis guidelines from 2013 suggested dobutamine infusion in the presence of myocardial dysfunction or ongoing signs of hypoperfusion despite achieving adequate intravascular volume and adequate mean arterial pressure^25^. As a surrogate of low cardiac output, we used mixed venous oxygen saturation which represents the body’s oxygen reserve by taking into account not only cardiac output, but rather systemic oxygen delivery in relation to oxygen consumption.

Blood glucose was maintained between 3.5 and 7.0 mmol/L by G50% or insulin infusion. Respiratory rate and minute ventilation were adjusted in order to maintain pH between 7.35 and 7.45. Arterial oxygen partial pressure was kept between 100 and 150 mmHg and oxygen saturation above 90%. PEEP and FiO2 were adjusted accordingly and tracheal suctioning as well as pulmonary recruitment manoeuvres were performed regularly. As in human abdominal sepsis, piperacillin-tazobactam (2.25 g/8 h iv; Sandoz Pharmaceuticals, Rotkreuz, Switzerland) was used as an antibiotic. Liquemin 10000 IU/24 h was infused IV to prevent deep vein thrombosis. If core temperature exceeded 39.5 °C, the animal was cooled by fan, lowering of room temperature, alcohol spray and ice bags. Further blood samples were taken just before RP + 24 h (T3), RP + 48 h (T4) and two of hours after RP + 72 h (T5). At the end of the experiment animals were euthanized by bolus infusion of 40 mmol of potassium chloride. Further details can be found in^26^.

### Hemodynamic analyses

All signals were continuously recorded for the entire duration of the experiment. The time points used for blood analyses were considered as reference to identify and select stationary portion of signals (5–10 min) for successive analyses. The carotid arterial blood pressure (ABP) and blood flow (CBF) were analysed and the onsets of each heart pulse were identified using available algorithms^27,28^. The following beat-to-beat time series were derived from ABP signals: systolic arterial pressure (SAP), diastolic arterial pressure (DAP), mean arterial pressure (MAP), pulse pressure (PP), and heart period (HP), defined as the time difference between consecutive onsets (surrogate for RR-intervals). Beat-to-beat mean CBF was also computed. Continuous cardiac output (CCO) and central venous pressure (CVP) were averaged on each HP time interval to get beat-to-beat series. Beat-to-beat values of carotid stroke volume (carotid SV) was assessed from CBF by integrating it over each heart cycle by means of the trapezoid method; beat-to-beat values of global stroke volume (global SV) were computed as $CCO/HR$. Finally, mean values of all time series were computed for each phase of the experiment.

### Autonomic cardiovascular indices

ANS cardiovascular control was evaluated by estimating the power spectral density of DAP component and the cardiac baroreflex sensitivity (BRS). The time series of DAP values were resampled at 2 Hz to obtain evenly spaced signals, successively were detrended using a 10th order polynomial function, and then normalized by dividing the detrended signal by its standard deviation in order to have zero-mean oscillations and unitary SD. The stationarity of the resulting time series was assessed by the Kwiatkowski-Phillips-Schmidt-Shin (KPSS) test. Power spectra were estimated via autoregressive spectral analysis. Spectral indices were computed as low-frequency power (LF, 0.04–0.15 Hz), total power (TP), which is the total area under the spectrum, and the normalized LF power, i.e. LF/(TP-VLF)%, where VLF is the very low frequency power [0–0.04 Hz].

The bivariate closed-loop model method^29^ was adopted to estimate the cardiac BRS on the time series of SAP and HP which were resampled at 2 Hz. We decided to restrict the analysis of BRS only to the LF component of the feedback gain, since this component gives indications on the sympathetic activity^30^. The autonomic cardiovascular indices were computed over 2-min windows with 50% overlap and then averaged.

### Windkessel vascular indices

The arterial tree was modelled as a two-element Windkessel model, and several indices were derived to characterize its properties. The Windkessel time constant $\tau$ is defined as $\tau =TPR*AC$, where TPR is the total peripheral resistance and AC is the total arterial compliance, it represents the exponential decay of ABP pulse from systolic peak to end of diastole^31^. The method proposed by Mukkamala and colleagues^32^ estimates the exponential decay over pretty large windows (6 min) so to avoid cumulative effects of the wave reflections, which are attenuated in the central ABP waveforms only. More specifically, the ABP response to a single, solitary cardiac contraction is estimated from the ABP waveform. Then, the Windkessel time constant is measured by fitting an exponential to the tail end of this estimated response once the faster wave reflections have vanished. We adopted this method for a robust estimate of $\tau .$

Arterial compliance is defined as the change in arterial blood volume due to a given change in arterial blood pressure^33^; we thus computed the beat-to-beat carotid arterial compliance (cAC) as $carotid SV/PP$ according to the definition. For clarity sake the CBF was measured in the right carotid while carotid ABP in the left carotid, we reasonably assumed that the changes and values were similar.

We calculated the vascular resistance, previously named TPR, with two different approaches based on the Windkessel model^31^. We named systemic vascular resistance (SVR) the estimate obtained with the global CCO, and carotid vascular resistance (carotid VR) the estimate obtained with the CO computed from the carotid SV (CO = SV*HP). Both vascular resistance indices were then normalized by the animal weight:

1 $$SVR = \frac{MAP - CVP}{{CCO}}/weight$$

2 $$carotid{ }VR = \frac{MAP - CVP}{{carotid{ }CO}}/weight$$

The indices of vascular resistance and cAC were computed on a beat-to-beat basis and then averaged at each time point. The index $\tau$ was computed over 6-min 50% overlapping time window and then averaged to get a single value at each time point.

### Effective arterial elastance

Effective arterial elastance (Ea) is considered a simple estimate of total arterial load. Monge Garcia et al.^34^ proposed an estimate of Ea from a blood pressure measurement recorded in a peripheral vascular district; they proved that, given a reliable SV measurement, the ratio MAP/SV provides a robust Ea surrogate over a wide range of hemodynamic conditions and is interchangeably in any peripheral artery. In this case MAP is proposed as a surrogate for the left ventricular end-systolic pressure (LV ESP). We similarly estimated Ea by using carotid artery pressure and carotid stroke volume according to Eq. (3). Carotid is a direct branch of aorta so we can suppose that this measure can provide reliable information of heart-vascular coupling and total cardiac afterload.

3 $$Ea = MAP/carotid{ }SV$$

Ea was computed on a beat-to-beat basis and then averaged at each time point.

### Therapy and laboratory data

The overall dosages of fluids and vasopressors (VP) were calculated by summing up the dosage from the start of the experiment to each time point. The only vasopressor administered was noradrenaline (100 μg/mL and 200 μg/mL), and the fluids used were NaCl 0.9%, ringer lactate and glucose solution (G50%). Urine output UO (mL) was retrieved as the cumulative amount from the start of the experiment until each time point. Fluid balance (mL) was computed as the difference between the amount of intravenous fluids given and UO at each time point. The amount of sedative drugs (propofol and fentanyl) was computed as cumulative dosage at each time point. VP, fluids, UO, fluid balance and sedation amount were normalized to the animal’s weight. We also considered the following parameters: mixed venous oxygen saturation (SvO2, %), hematocrit (Htc, L/L), hemoglobin (Hb, g/L), platelets (Plat, 10^9^/L), leukocytes (WBC, 10^9^/L), creatinine (Creat, µmol/L), prothrombin time (PT, sec), bilirubin (µmol/L), ammonia (µmol/L), and band neutrophils (N%, %). Finally, high sensitive cardiac troponin I (hs-cTnI, ng/L) was measured from plasma samples using a specific ELISA kit (CTNI-9-US, Life Diagnostics) to have an indirect measure of cardiac stress.

### Statistical analysis

Wilcoxon rank-sum test was adopted to verify significant differences in the indices between the two groups at each time point. Friedman test was adopted to find differences within each group among time points across multiple tests attempts. In case Friedman test *p*-value < 0.05, we used then the Wilcoxon signed-rank test to assess significant changes among pairs of time points within each group of animals. For the post-hoc multiple comparisons we adopted the Tukey’s honestly significant difference correction for p-value significance. Significance was considered with a *p*-value < 0.05.

## Results

Four pigs out of 20 were excluded from the original population since the quality of collected signals was not guaranteed for all the time points and the artefact-free segments were too short due to technical reasons. The resulting dataset was composed by 9 pigs in the septic group and 7 in the sham group.

The measurement of CCO at time point T2 was not available for the majority of the septic animals due to technical reasons, i.e. unavailable acquisition due to hyperthermia during sepsis development, therefore CCO and derived indices (i.e. global SV, Ea, SVR) at T2 were excluded from the statistical analyses.

### Clinical parameters and hemodynamic indices

**Figure 1:**
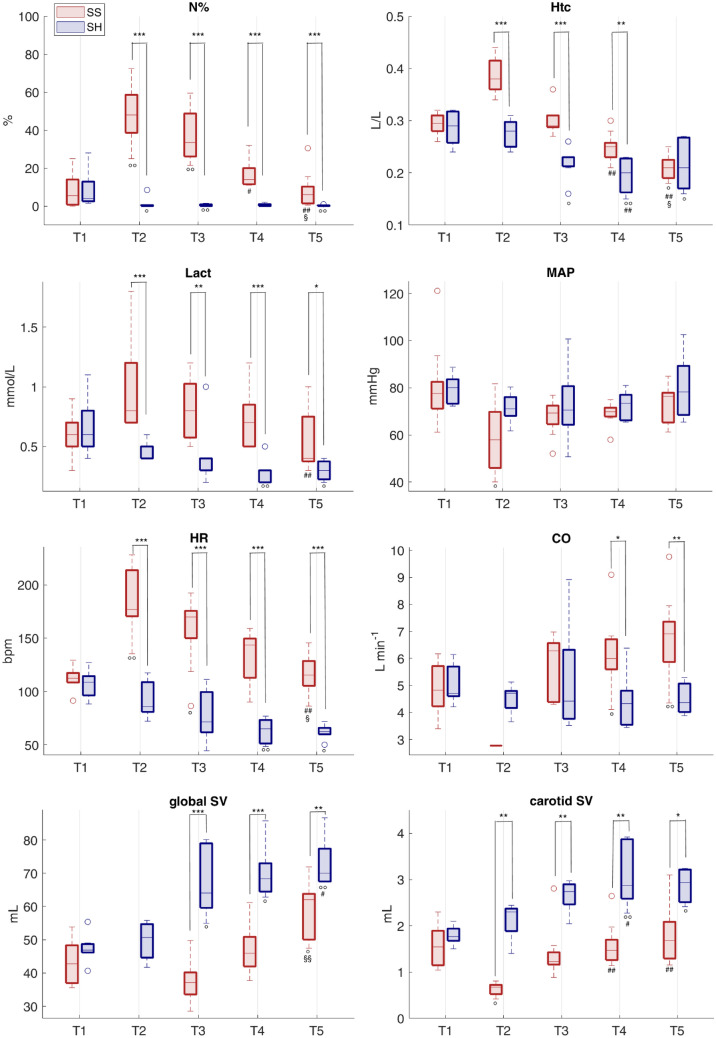
Distributions of laboratory data and hemodynamic variables for the two groups at each time point. Comparison between Sepsis and Sham: *p-value < 0.05, **p-value < 0.01, ***p-value < 0.001 (Wilcoxon rank sum test). Wilcoxon signed test: °*p*-value < 0.05, °°*p*-value < 0.01 with respect to T1, #*p*-value < 0.05, #﻿#*p* < 0.01 with respect to T2, §*p*-value < 0.05, §﻿§*p*-value < 0.01 with respect to T3 (Friedman test *p*-value < 0.05). N% = band neutrophils, Htc = hematocrit, Lact = arterial lactate, MAP = mean arterial pressure, HR = heart rate, CO = cardiac output, global SV = global stroke volume, carotid SV = carotid stroke volume.

Table 1 and Fig. 1 report the laboratory, hemodynamic and therapy data of septic and sham pigs at all time points. At baseline, all animals present a similar condition in terms of both hemodynamic profile and laboratory variables. After 6–8 h from inoculation of feces, SS animals showed clear signs of sepsis (time point T2 in Fig. 1 and Table 1); a marked increase in band neutrophils percentage confirmed the inflammation; the inflammatory response resulted in an increased endothelial permeability and capillary leakage represented by a significant increase in hematocrit and hemoglobin values at T2 (Fig. 1 and Table 1); moreover, these indices were significantly higher in SS with respect to SH group. Tissue hypoperfusion is suggested by the significant reduction in blood pressure and SV, together with an increase in serum creatinine and ammonia. Lactate concentration was significantly higher in septic animals than in controls, but remained below 2 mmol/L (Fig. 1 and Table 1). However, due to species differences with humans, normal lactate levels are usually observed in swine septic shock^35^ despite developing all the criteria for a septic shock and tissular hypoperfusion^36^. According to the surviving sepsis campaign guidelines from 2013^25^, which were the only available at the time this protocol was designed, severe sepsis was defined as sepsis plus sepsis-induced organ dysfunction or tissue hypoperfusion; our porcine sepsis phenotype is therefore compatible with this definition. Moreover, the hypotensive state together with the systemic inflammation triggered a tachycardic condition in SS pigs, that was characterized by a significantly higher HR compared to SH at T2 (Fig. 1 and Table 1). Unfortunately, CCO and global SV were not available at T2; however, CBF was significantly decreased in SS pigs, together with the computed carotid SV, hinting a global decrease in systemic CO (Fig. 1 and Table 1). Finally, the significantly higher values of creatinine, bilirubin and ammonia in SS pigs compared to SH, may indicate an initial hepatic and renal dysfunction (Table 1).

**Table 1:** Medians (25–75th percentiles) values of the laboratory analyses, hemodynamic measures, and clinical and therapy data at each time point for the two groups (septic pigs SS n = 9, sham pigs SH n = 7, if not stated otherwise in the table).

		T1	T2	T3	T4	T5
**Laboratory analyses**						
SvO2	SS	57.3 (53,62.8)	46 (42,56.3)	63.5 (61.4,67.9)##	66.6 (62.6,67.2)##	60.3 (59.4,65)#
	SH	57 (52.4,61.3)	53 (50,53.8)	56 (50,66.8)	55.1 (52.1,57.8)**	56.7 (53.1,63.5)
Htc	SS	0.29 (0.28,0.31)^n=8^	0.38 (0.36,0.41)	0.29 (0.29,0.31)	0.25 (0.23,0.26)#	0.21 (0.19,0.23)°#§
	SH	0.29 (0.26,0.32)	0.28 (0.25,0.30)*	0.23 (0.21,0.23)*°	0.2 (0.16,0.23)*°#	0.21 (0.17,0.27)°§
Hb	SS	101 (91,102)	132 (119,141)	100 (94,106)	83 (76,89)#	71 (62,78)°#§
	SH	98 (86,106.2)	95 (84.0,98)*	73 (69.8,79)*°	66 (52.5,76.8)*°#	70 (56.5,87)°
Plate	SS	276 (244,445)^n=8^	192 (174,212)	85 (58,94)°#	102 (68,112)°	142 (107,166)
	SH	231 (208,306)	193 (158,229)	151 (136,187)*°	174 (156,198)*°	223 (213,237)*§
WBC	SS	19.2 (16.2,23.3)^n=8^	11.7 (9.7,13.1)	11.5 (10.6,17.6)	11.5 (9.6,13.6)°	10.2 (8.3,12.1)°
	SH	21.4 (18.6,26.2)	18.3 (13.1,20.8)*	13.8 (11.1,19.2)	13.1 (9.6,14)°	11.9 (10.1,15.4)°
PT	SS	13.9 (13.2,14.3)^n=8^	14.7 (14.6,16.2)	14.7 (14.1,15.1)	14.2 (13.6, 14.7)	14.1 (13.7,14.9)
	SH	13.9 (13,14.1)	14.1 (13.8,14.5)*	14.3 (13.2,14.5)	13.5 (13,13.7)	13.7 (13.4,13.7)*
N%	SS	5.5 (0.8,14)^n=8^	48 (38.6,58.6)°	33.5 (26.1,48.8)°	14 (11.5,20)#	6 (1.4,10.2)#§
	SH	4 (2.5,12.9)	0 (0,0.5)*°	0 (0,0.9)*°	0.5 (0,1.0)*	0 (0,0.4)*°
Lact	SS	0.6 (0.5,0.7)	0.8 (0.7,1.2)	0.8 (0.6,1)	0.7 (0.5,0.9)#	0.4 (0.4,0.8)
	SH	0.6 (0.5,0.8)	0.4 (0.4,0.5)*	0.3 (0.3,0.4)*	0.3 (0.2,0.3)*°	0.3 (0.2,0.4)*°
Creat	SS	94.0 (86.8, 100.2)	174 (150.8,182.5)°	113 (106.5,165.2)	99 (87.2,112)	89 (80.5,96.2)#§
	SH	92 (76.8,100)	106 (98,111.5)*	85 (79.5,97.8)*	86 (65,95.2)#	84 (69.5,86.8)#
Bilirubin	SS	1.2 (0.7,1.6)	1.4 (1.1,2.4)	1.5 (1.3,1.9)	1.3 (0.8,2.5)	1.4 (0.9,3.6)
	SH	1.1 (0.6,2)	0.9 (0.6,1.6)	0.8 (0.4,1.9)	0.5 (0.3,0.8)*	0.8 (0.3,1.1)
Ammonia	SS	51 (36.8,66.5)	107 (72,125.2)^n=8^°	85 (67,94.5)^n=8^	78 (61.2,88)	95 (88,114)°
	SH	51 (48.2,53.5)	35 (29,36)^n=6^ *°	42.5 (38,50)^n=6^ *	43 (40.8,52.8)*	54 (53,66.5)*#§
**Hemodynamic variables**						
SAP	SS	103 (94,107)	72 (65,95)°	101 (93,106)	96 (91,102)	97 (96,109)#
	SH	103 (97,107)	100 (94,103)*	103 (90,109)	99 (91,106)	99 (94,118)
DAP	SS	59.6 (53.2,68.6)	43.8 (34.9,52.7)°	47.5 (44.7,54.1)°	51.1 (48.1,53.9)	57 (50.4,60.5)
	SH	59.1 (57.8,64.9)	49.3 (47.4,58.5)	53.3 (43.1,58.9)	54.4 (46.6,57.1)	58.2 (48.3,68)
MAP	SS	77.6 (71.1,82.5)	57.9 (46,69.8)°	69.3 (64.6,72.4)	69.8 (67.8,71.5)	76.4 (65.3,77.9)
	SH	80.1 (73.2,83.6)	71.1 (68.1,76)	70.5 (64.3,80.7)	73.4 (66.2,77)	78.2 (68.5,89.3)
PP	SS	43.0 (35.7,46.9)	32 (29.5,34.4)	49.9 (42.8,55.7)#	46.6 (40.7,51.5)#	48.9 (36.5, 52.6)
	SH	44.3 (32.7,46.5)	48.4 (38.4,54.3)*	40.3 (38.0,47.3)	46 (43.3,54.8)	45.5 (41.6,50.5)
HR	SS	112 (108,117)	177 (171,214)°	170 (150,176)°	144 (113,150)	115 (105,129)#§
	SH	109 (96,114)	86 (81,109)*	72 (62,100)*	65 (51,73)*°	62 (60,66)*°
CVP	SS	5.5 (4,6.4)^n=8^	5.6 (3.1,5.9)^n=8^	6.3 (3.3,8.5)	7.6 (6.5,9.9)#	10.9 (8,12.1)°#
	SH	5.4 (4.2,6.3)	5.9 (4.9,6.6)	8.1 (6.4,9.1)	7.1 (6.6,8.6)	7.2 (6.1,8.6)
CCO	SS	4.8 (4.2,5.7)^n=8^	–	6.3 (4.4,6.6)	6 (5.6,6.7)°	6.9 (5.9,7.4)°
	SH	4.7 (4.6,5.7)	4.7 (4.2,4.8)	4.4 (3.8,6.3)	4.3 (3.5,4.8)*	4.4 (4,5.1)^n=6^ *
CBF	SS	176 (133,204)	108 (101,124)	215 (171,241)#	191 (180,235)#	171 (154,235)#
	SH	182 (168,204)^n=5^	181 (167,187)^n=5^ *	184 (160,214)^n=6^	179 (167,195)^n=6^	185 (148,198)^n=6^
Global SV	SS	42.7 (36.9,48.3)^n=8^	–	37.1 (33.5,40.2)	46 (42,50.9)	62 (50,63.8)°§
	SH	46.9 (46.2,48.7)	50.7 (44.6,54.7)	64.1 (59.6, 79)*°	68.3 (64.5,73)*°	70 (67.5,77.4)^n=6^ *°#
Carotid SV	SS	1.54 (1.15,1.89)	0.67 (0.52,0.72)°	1.23 (1.16,1.43)	1.47 (1.26,1.7)#	1.68 (1.29,2.09)#
	SH	1.77 (1.68,1.94)^n=5^	2.3 (1.89,2.37)^n=5^ *	2.74 (2.46,2.90)^n=6^ *	2.87 (2.59,3.87)^n=6^ *°#	2.94 (2.51,3.21)^n=6^ *°
**Clinical and therapy data**						
VP	SS	0 (0,0)	0 (0,0)	264.2 (120.9,1503.7)	509.7 (198.4,3181)	595.5 (217.4,4341.5)
	SH	0 (0,0)	0 (0,0)	0 (0,9.7)**	0 (0,17.2)**	0 (0,21.1)**
Fluids	SS	46 (40.4,51)	59.9 (55.1,64.3)	169 (162.3,177.3)	242.7 (232.5,250.7)	325.5 (284.3,332.5)
	SH	60.2 (35,71.4)	74.7 (48.1,83)	149.1 (123.7,168.3)	223.4 (191.8,237.7)	271.3 (249.9,298.3)
Propofol	SS	75.7 (63.3,89.4)	160.8 (131.4,176.1)	392 (326.8,411.5)	623.7 (514.7,642.8)	888.4 (739.7,913)
	SH	68.4 (59,73.6)	140.6 (120.8,150.5)	367.1 (345.4,371.9)	590.6 (544.7,612.4)	842.5 (804.2,1005.2)
Fentanyl	SS	37.6 (34,62.6)	82.6 (63.6,169.1)	210.3 (166.8,442.2)	316.3 (264.6,711.5)	468.6 (401.8,1016.4)
	SH	42.7 (35.6,45.9)	80.7 (68.3,87.5)	203.9 (184.8,212.5)	283.7 (273.8,351.3)	399.3 (372.1,468)
UO	SS	25.2 (22,31)	32.4 (29.9, 37.4)	58.6 (53.7, 65.9)	87.5 (76.3, 92.5)	103.5 (99.3, 112.5)
	SH	27.7 (23.2, 31.9)	37.8 (33.5, 42.5)	68.8 (66.4, 75.6)	102.9 (90.0, 115.8)	142.5 (125.1, 172.5)*
FB	SS	19.4 (10.2,28.3)	23 (19.4,32.6)	110.4 (100.4,122.5)	159.4 (150,167.6)	209.7 (181,227.8)
	SH	37.4 (9,42.4)	35.7 (12.8,42.2)	79.7 (53.7,98.1)	120.8 (72.7,146.4)	118.1 (66.7,175.6)

SvO2 = mixed venous oxygen saturation [%], Htc = Hematocrit [L/L], Hb = Hemoglobin [g/l], Plat = platelets [10^9^/L], WBC = white blood cells [10^9^/L], PT = prothrombin time [sec], N% = Band neutrophils [%], Lact = lactate [mmol/L], Creat = Creatinine [μmol/L], Bilirubin [μmol/L], Ammonia [μmol/L], SAP = systolic arterial pressure [mmHg], DAP = diastolic arterial pressure [mmHg], MAP = mean arterial pressure [mmHg], PP = pulse pressure [mmHg], HR = heart rate [bpm], CVP = central venous pressure [mmHg], CCO = continuous cardiac output [L/min], CBF = carotid blood flow [mL/min], global SV = global stroke volume [mL], carotid SV = carotid stroke volume [mL], VP = cumulative amount of vasopressors [μg/Kg], Fluids = cumulative fluid input [mL/Kg], cumulative amount of Propofol [mg/Kg], cumulative amount of Fentanyl [µg/Kg], UO = cumulative urine output [mL/Kg], FB = Fluid balance [mL/Kg].

Comparison between Sepsis and Sham: *p-value < 0.05, **p-value < 0.01 (Wilcoxon rank sum test). Wilcoxon signed test: °*p* < 0.05 with respect to T1, #*p* < 0.05 with respect to T2, ##*p* < 0.01 with respect to T2, §*p* < 0.05 with respect to T3, ^*p* < 0.05 with respect to T4 (Friedman test *p*-value < 0.05). The number reported in the apex refers to the number of pigs with available data, if not all.

**Figure 2:**
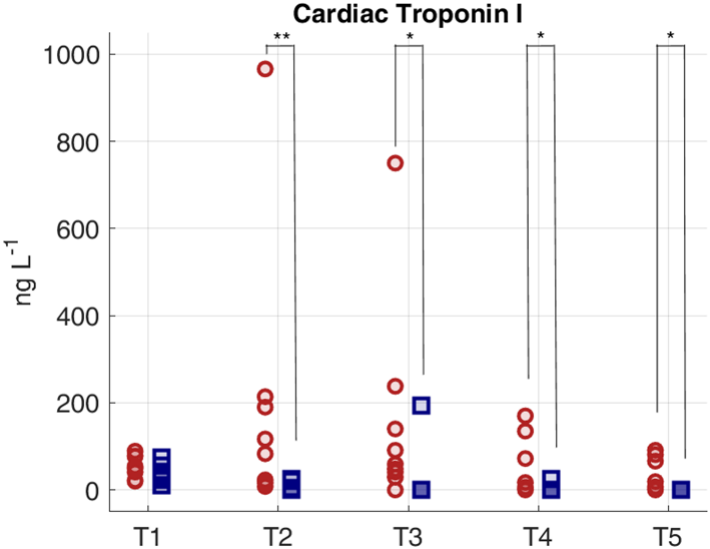
Values of high sensitive cardiac troponin I (hs-cTnI) concentration in arterial plasma at each time point of the experiment for both populations. Red circles represent septic animals, blue squares represent sham animals. Comparison between Sepsis and Sham: *p-value < 0.05, **p-value < 0.01 (Wilcoxon rank sum test).

The main targets of hemodynamic resuscitation, according to international guidelines, is to restore a MAP ≥ 65 mmHg by means of fluids and vasopressors therapy and to maintain a sufficient blood oxygenation for organ perfusion. The resuscitation protocol was effective in restoring and keeping MAP in the target range and to significantly increase SvO2 which was maintained above 60% in almost all animals (Fig. 1, Table 1). However, we found that the tachycardic response did not resolve in the 24 h after resuscitation and, although the HR decreased, SS group maintained significantly higher values than SH group for the next three days until the end of the experiment (Fig. 1). Besides, at T4 and T5, SS group showed a significant increase in the values of CO with respect to baseline, and the values were significantly higher with respect to SH group (Fig. 1). SV was instead lower in SS pigs with respect to SH (Fig. 1). This condition of persistent tachycardia can be a source of cardiac stress, as documented by the significantly increased concentration of cardiac troponin I in the arterial plasma of septic animals with respect to sham until the end of the experiment (Fig. 2).

**Figure 3:**
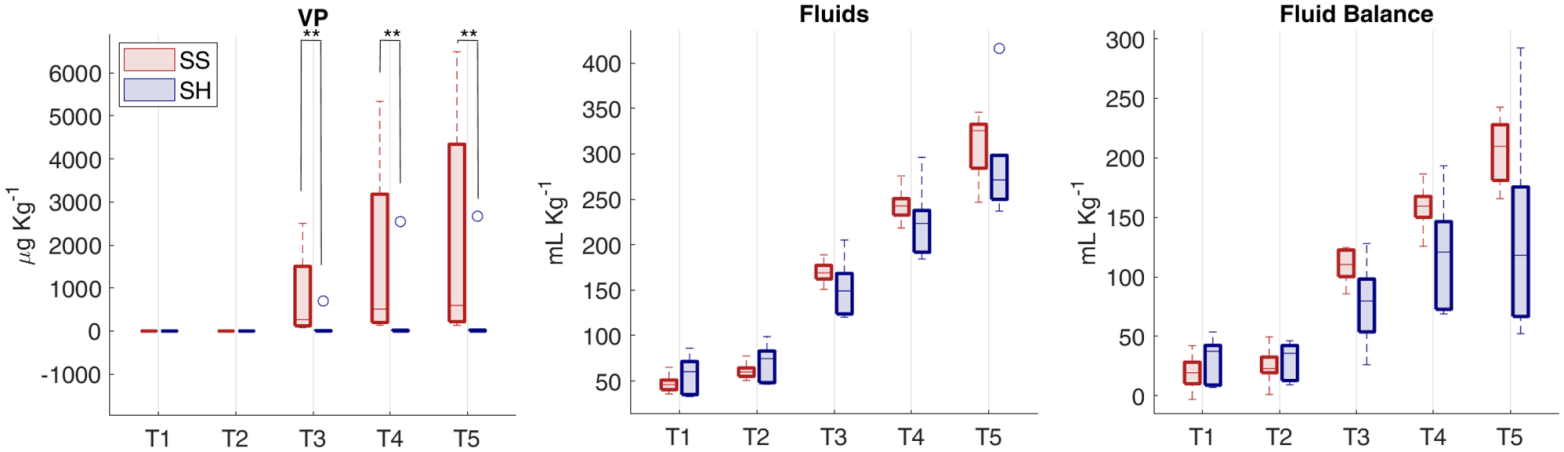
Distributions of administered vasopressors (VP), fluids and fluid balance for the two groups at each time point. Indices are computed as cumulative values from the start of the experiment until each time point and are normalized with respect to each pig’s weight. Comparison between Sepsis and Sham: **p-value < 0.01 (Wilcoxon rank sum test).

Figure 3 shows the distribution of vasopressor dosage and fluids input normalized by pig’s weight, both computed as cumulative values from the start of the experiment until each time point. As expected, SS pigs received significantly higher dosages of VP; moreover, the fluid balance resulted higher in SS group, due to the decreased urine output (significant at T5, Table 1) and the slightly higher amount of fluids administered.

### Cardiovascular autonomic control

Sepsis induced a significant drop in BRS (time point T2, Table 2), hinting a failure of autonomic control of the cardiovascular system. To note, this impaired condition seems not resolved after resuscitation as SS group maintained significantly lower values of BRS with respect to SH group until the end of the experiment, and the BRS values in septic pigs were slightly lower than baseline condition even at T5, i.e. after 3 days of treatment. Moreover, at T2, SS group showed a reduction in LF power of DAP series with respect to baseline values (Table 2). DAP can be seen as a surrogate of systemic vascular resistance, therefore, this result might indicate a decreased sympathetic outflow to the vasculature, probably associated to the vasodilation triggered by sepsis. Noteworthy, the dosage of sedative drugs was similar between septic and sham animals (Table 1), despite a larger range of fentanyl in septic animals (electronic supplement Fig. S1); this allows to exclude sedation as important factor affecting the different autonomic response in the two groups of animals.

**Table 2:** Medians (25–75th percentiles) values of cardiac baroreflex sensitivity and power spectral indices of diastolic arterial pressure time series computed at each time point for the two groups (septic pigs SS n = 9, sham pigs SH n = 7).

		T1	T2	T3	T4	T5
BRS	SS	3.1 (2.3,4.4)	0.8 (0.4,1.5)°	1.9 (1.2,2.8)	1.9 (1.4,4.9)	1.9 (1.1,5)
	SH	2.4 (1.6,3.4)	5.3 (2.7,7.7)*	5.2 (3.7,6.4)*	4.7 (2.8,8)	7.8 (4.6,8.5)*
LF DAP	SS	308 (274,337)	97 (51,172)°	147 (75,277)	182 (133,299)	213 (185,299)
	SH	296 (164,456)	290 (229,351)*	213 (180,295)	181 (173,231)	227 (178,346)
LFu DAP	SS	59.7 (55.9,74.3)	21.9 (10.5,33.8)°	24 (16.6,60.8)	38.9 (27,65.5)	55.2 (39.6,62.3)
	SH	61.7 (33.8,89.3)	59.4 (46.8,71)*	43.7 (36.6,63.2)	40.3 (36.4,48.8)	43.8 (37.5,70.8)
TP DAP	SS	521 (514,534)	610 (501,771)	527 (508,535)	526 (501,545)	542 (505,547)
	SH	561 (503,595)	531 (519,555)	532 (522,546)	526 (515,546)	539 (515,552)

BRS = baroreflex sensitivity [ms/mmHg], LF DAP = low frequency power of diastolic blood pressure [a.u.], LFu DAP = normalized low frequency power of diastolic blood pressure [%], TP DAP = total power of diastolic blood pressure [a.u.].

Comparison between Sepsis and Sham: *p-value < 0.05 (Wilcoxon rank sum test). Wilcoxon signed test: °*p*-value < 0.05 with respect to T1 (Friedman test *p*-value < 0.05).

### Vascular alterations

**Figure 4:**
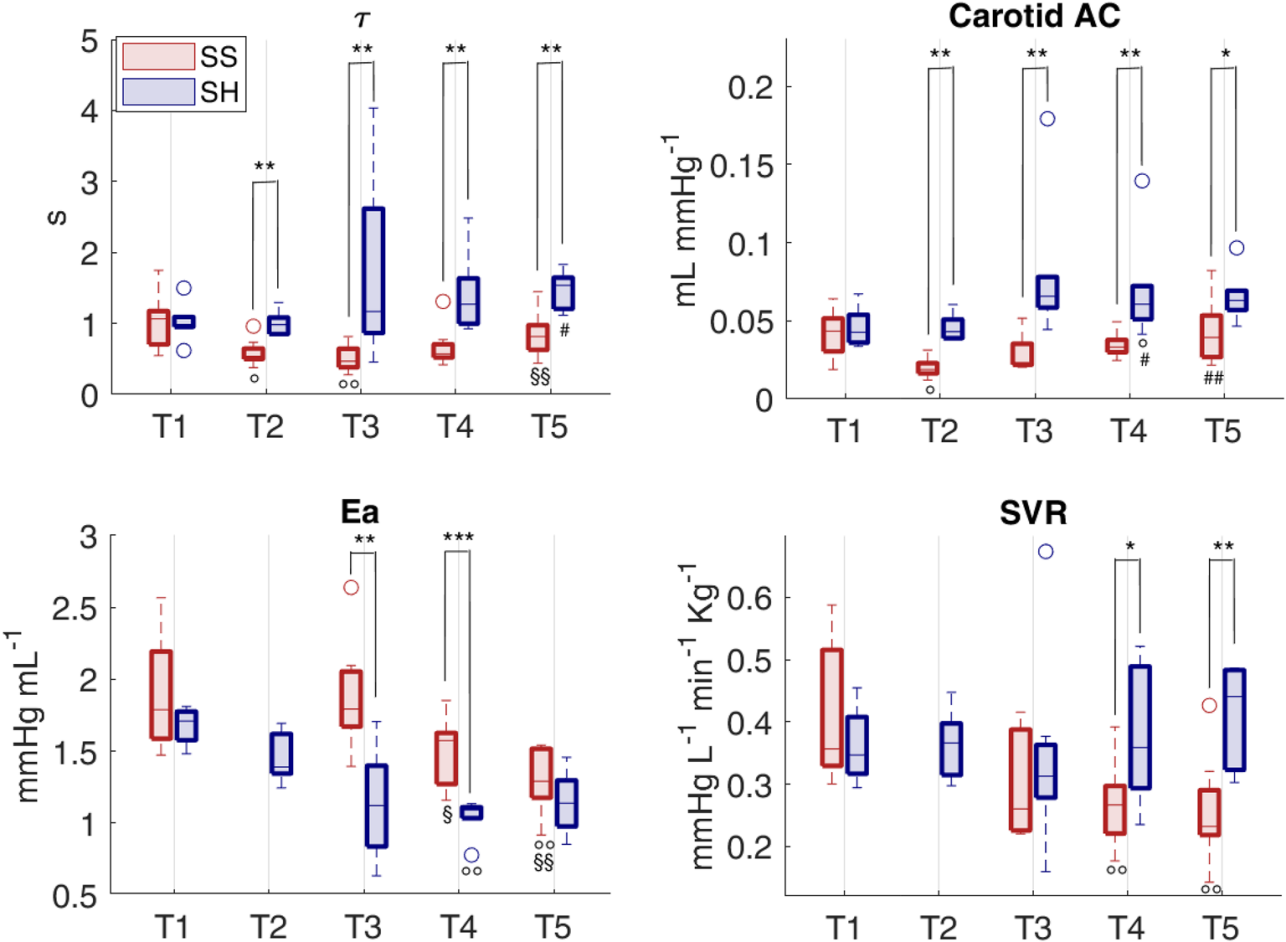
Distributions of values of vascular indices for the two groups at each time point. Comparison between Sepsis and Sham: *p-value < 0.05, **p-value < 0.01, ***p-value < 0.001   (Wilcoxon rank sum test). Wilcoxon signed test: °*p*-value < 0.05, °°p-value < 0.01 with respect to T1, #*p*-value < 0.05, ##*p*-value < 0.01  with respect to T2, §*p*-value < 0.05, §§*p*-value < 0.01 with respect to T3 (Friedman test *p*-value < 0.05). τ = characteristic arterial tree time constant, cAC = carotid arterial compliance, Ea = effective elastance, SVR = systemic vascular resistance.

**Figure 5:**
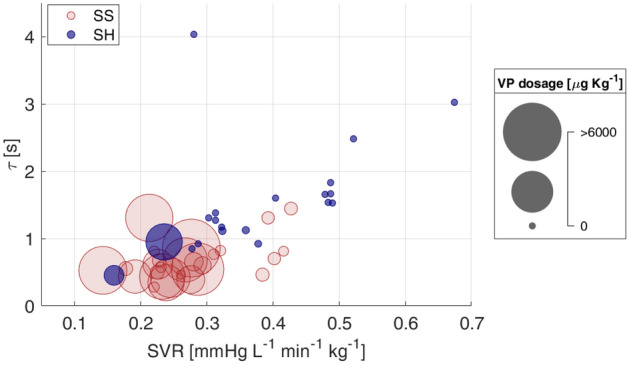
Bubble plot highlights possible relations between therapy and vascular indices. Each bubble refers to a pig at T3, T4 or T5 and the radius is proportional to vasopressor dosage.

The values of the Windkessel time constant τ and cAC were reduced after sepsis development in SS group and remained lower than those of sham group until the end of the experiment (Fig. 4). Higher Ea values were found in SS group compared to SH group during the entire resuscitation phase, meaning an elevated afterload (Fig. 4). Finally, the values of carotid VR were significantly higher in SS group compared to SH group at T2 (Table 3). In the next three days of resuscitation, instead, SS group was characterized by a lower SVR (Fig. 4); in particular, the values of SVR were significantly lower in SS group with respect to SH group at T4 and T5 and to baseline values. Figure 5 shows the relationship between vascular properties and vasopressor therapy in terms of SVR and $\tau$ values together with VP dosage normalized by each pig’s weight. As expected, SS pigs received significantly higher dosages of VP, although they exhibited relatively low SVR compared to sham, which, on the contrary, displayed higher values of SVR and Windkessel time constant $\tau$, and most of them received no vasopressor. Only two sham pigs received noradrenaline, one developed systemic inflammatory response syndrome (SIRS) during the experiment, the other went into respiratory failure at day 4. From these results septic pigs may be considered less responsive to VP therapy in terms of vascular resistance, and this condition may be related to a modification of arterial tree characteristics and mechanical properties induced by sepsis; in these terms the therapy was ineffective in restoring the basal condition.

**Table 3:** Medians (25–75th percentiles) values of carotid vascular resistance computed at each time point for the two groups (septic pigs SS n = 9, sham pigs SH n = 7, if not stated otherwise in the table).

		T1	T2	T3	T4	T5
Carotid VR	SS	10.5 (9.1,13.3)^n=8^	11.9 (10.8,14.7)^n=8^	7.4 (6.3,9.9)	8.3 (7.1,9.1)°#	9.3 (7.3,10.8)
	SH	9.5 (8.8,10.8)^n=5^	8.8 (8.6,9.8)^n=5^ *	8 (7.6,8.7)^n=6^	8.7 (8.1,9.6)^n=6^	10.7 (8.1,10.9)^n=6^

Carotid VR = carotid vascular resistance [mmHg/mL/min/Kg], Comparison between Sepsis and Sham: *p-value < 0.05 (Wilcoxon rank sum test). Wilcoxon signed test: °*p* < 0.05 with respect to T1, #*p* < 0.05 with respect to T2 (Friedman test *p*-value < 0.05).

## Discussion

In this study we evaluated the effect of sepsis development and protocolled resuscitation on autonomic mechanisms for cardiovascular control and arterial vascular properties during a 5-day swine experiment. Our results generally confirmed previous studies^22,37^, highlighting a persisting autonomic failure and vascular disarray despite successful resuscitation, as defined by the hemodynamic and clinical targets suggested by the international guidelines (e.g. MAP > 65 mmHg)^38^.

As expected, sepsis induced a severe inflammation, hypoperfusion, hypotension, hypovolemia and tachycardia, as shown in Fig. 1 and Table 1. The resuscitation manoeuvres, including antibiotics, were able to restore MAP > 65 mmHg and SvO2 > 60%, to reduce inflammation, and to increase the circulating volume as the CO values increased. While these changes can be considered signs of therapy effectiveness, we observed a persistent tachycardia in SS pigs until the end of the experiment, i.e. after three days of therapy administration. Tachycardia, typically triggered by sepsis, is the main compensatory mechanism to maintain a sufficient CO despite reduced SV either due to reduction of preload or to diastolic and/or systolic dysfunction. However, even after adequate fluid resuscitation to correct for hypovolemia, septic patients often show an elevated HR, and this condition has been demonstrated to be associated to higher mortality, incidence of septic cardiomyopathy and cardiac dysfunctions^9,13,39^. Indeed, the raised cardiac troponin I observed in septic pigs (Fig. 2) indicate an elevated stress of the cardiomyocytes, which can further worsen and lead to severe dysfunctions if long lasting^40^. For this reason, several preclinical and clinical studies have investigated new drugs able to attenuate this persistent tachycardia in order to improve cardiac functionality and the clinical outcome^41,42^. Despite a lack of guidelines recommending lowering heart rate, very promising results have been achieved by administering cardioselective β1-blocker drugs, such as esmolol, in large randomized clinical trials^43^. Recent studies have hypothesized an anti-inflammatory action exerted by esmolol, decreasing lactate and proinflammatory cytokines concentrations, but also improving vascular responsiveness to vasopressors^44,45^.

**Figure 6:**
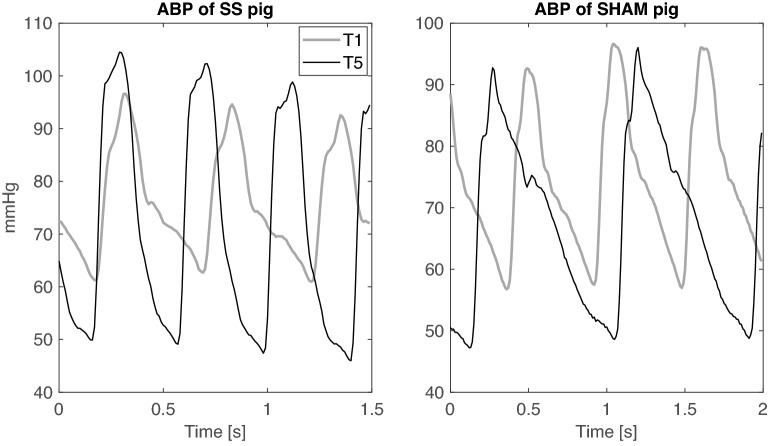
Example of carotid arterial blood pressure waveform of a septic pig and a sham pig, at T1 (baseline) and T5 (72 h after the start of resuscitation).

Acute inflammation and sepsis are known to impair endothelial functions and to induce a stiffening of large compliant vessels, such as the aorta^16^. Our results confirmed this evidence as the values of the carotid compliance decreased after sepsis development (cAC T2, Fig. 4); similarly, the values of arterial time constant τ were significantly reduced. The present work demonstrates that hemodynamic resuscitation was able to restore neither τ nor carotid compliance when compared to sham animals after 3 days of therapy (T5). Moreover, SVR was also found to be significantly reduced in SS pigs with respect to SH after 48 h and 72 h of resuscitation (T4 and T5, respectively), despite increasing dosage of noradrenaline (Fig. 3). These vascular alterations can be observed from the graph of carotid ABP waveform (Fig. 6). The ABP morphological characteristics in a sham pig at T5 are similar to baseline (T1), on the contrary, the ABP waveform in a septic pig is completely different from baseline and SH pig, in particular, a much steeper decay of the diastolic phase, consistently with the lower values of τ and cAC previously described. In a previous study of our group^22^, we have reported a similar decreasing trend of τ and total arterial compliance in a septic shock swine population undergoing hemodynamic resuscitation with fluids and noradrenaline; in that case, the observation window was very short, about 5–6 h after therapy, and the insult was more severe (MAP reached values < 50 mmHg). All together these results point out that the vascular alterations occurring in the early phase of sepsis development characterize not only the first hours of therapy administration, when recovery from hypotension is of primary importance and massive fluids and vasopressors infusion are crucial for survival, but they persist also after days, when hemodynamic stabilization of the patient has been achieved. Long-term post-ICU disabilities are known to occur commonly in sepsis survivors, leading to a higher risk for future cardiovascular adverse events or hospital readmission^46^. Our results support the hypothesis that the persisting arterial stiffening plays a major role in this process, and a timely intervention to limit this phenomenon could positively impact the long-term outcome of septic patients. For example, in a previous experimental study^23^ we observed an improvement in compliance and τ values when septic shock pigs were treated with the adjunction of esmolol in comparison to septic pigs treated with fluids and noradrenaline only. The potential anti-inflammatory effect of esmolol together with its adrenergic antagonist effect could explain the improvement of vascular properties, as the proposed indices showed.

The increase in arterial stiffness may also help to explain the higher cardiac afterload in SS pigs compared to SH, measured from the effective elastance Ea index. In the original publication of Sunagawa et al.^47^, Ea was proposed as a steady-state arterial parameter that expresses all the extracardiac forces opposing to ventricular ejection, and incorporating peripheral resistance, characteristic impedance, total lumped arterial compliance and also systolic and diastolic time intervals; therefore, it is a net measure of ventricular afterload or arterial load. The gold-standard method to estimate Ea consists in the left ventricular pressure–volume analysis and Ea is estimated as the ratio of left ventricular end-systolic pressure (LV ESP) and stroke volume. However, given the increasing interest in using this index in critical care medicine, surrogates for LV ESP have been proposed and validated, in particular, from commonly available measures such as arterial peripheral pressure, in this study LV ESP was approximated with the mean arterial pressure^34^.

Higher values of Ea indicates higher “load” against which the heart must eject blood. This may lead to ventriculo-arterial (VA) uncoupling, depending on the cardiac reserve (cardiac preload and ventricular contractility)^48^. Several studies have reported that septic patients may often exhibit VA uncoupling^49,50^. Generally, uncoupling reflects a reduction in LV ejection efficiency, which can promote LV energetic failure and a condition of overall cardiovascular inefficiency. In the present study the LV elastance was not available, so we could not compute VA coupling. We can only speculate about possible mechanisms explaining the reduction in SV observed in SS pigs: it could be the result of a cardiac inability to cope with the elevated afterload (e.g. due to inotropic dysfunction), and tachycardia could thus reflect a compensatory mechanism in order to increase CO to meet the metabolic needs. On the contrary, reduced SV could be secondary to the elevated HR, the latter caused by autonomic chronotropic dysfunction. However, our data clearly indicates that 3 days of therapy administration were not able to restore the hemodynamic baseline condition in SS pigs, hinting that the overall cardiovascular system has been altered.

The administration of noradrenaline is expected (i) to induce vasoconstriction of arterial blood vessels, which usually causes a rise in blood pressure and vascular resistance; (ii) to induce an increase in preload due to venoconstriction and a redistribution of blood from unstressed to stressed volume; this leads to an increase in stroke volume and, consequently, in cardiac output; (ii) to increase cardiac contractility but to only minimally affect heart rate, i.e. initial transient increase through β1-receptors stimulation followed by a decrease due to activation of baroreceptors and vagal-mediated slowing of heart rate in response to the elevation in arterial pressure^51,52^. In our study, SVR was low in SS pigs, despite substantial amounts of vasopressor infused during the experiment, as shown in Fig. 5; moreover, the HR was significantly higher and the SV significantly lower in SS pigs compared to sham, as shown in Fig. 1. This highlights that other phenomena induced by the septic condition may interfere with the response to vasopressor administration, such as, e.g., baroreceptors dysfunction, adrenergic receptors desensitization or local release of vasoactive substances in the vessels^53^, and suggests that the overall hemodynamic response to sepsis therapy is highly dependent on the complex interaction between triggered inflammatory processes and drug therapy. In particular, the elevated burden of adrenergic stress derived from massive administration of noradrenaline may contribute to increased vascular stiffness, arterial afterload and HR. This prolonged adrenergic stress condition has been associated with increased overall mortality risk^6,10,54^. Moreover, large scientific evidence has reported a decreased arterial compliance in response to a sympathetic stimulation^19,55–57^, and in a recent clinical observational study^58^, elevated doses of noradrenaline were found to increase arterial characteristic impedance, pulse wave velocity (a measure of arterial stiffness), and reflection phenomena, while reducing aortic compliance. Conversely, reduced arterial compliance may interfere with the autonomic baroreflex mechanism by mechanically reducing baroreceptors sensitivity. We reported a decrease in carotid compliance, so we can reasonably suppose that the carotid baroreceptors may have been significantly affected. The loss of baroreceptors sensitivity is a potential mechanism explaining the reduced BRS observed in SS pigs compared to SH (Table 2). Another explanation could be the reduced responsiveness of the target organ, i.e. the heart: the adrenergic over-stimulation or, on the contrary, the suppressed parasympathetic activity at the heart level may prevent any further modulation of the HR by the ANS in response to pressure changes. The exact mechanisms responsible for these persistent cardiovascular alterations are not completely understood but they likely include a complex and multiscale interplay among different organs, i.e. the heart, the circulatory system, and the autonomic system. The potential of the proposed indices lies in the fact that they may capture the macroscopic phenotype of possible cellular events occurring in the organs’ tissues, and this may give an indication of the real effectiveness of the therapy, which may be overestimated when only global hemodynamic end-points are considered.

### Limitations of the study

The use of noradrenaline for hypotension guided by the SSC guidelines and dobutamine for low mixed venous oxygen saturation without documentation of hypoperfusion, i.e., hyperlactatemia, in this model could be challenged. Despite the SSC recommendation, the evidence that supports a specific mean arterial pressure target is weak. In fact, the most recent guidelines recommend a specific blood pressure target if the patient is on a vasopressor agent, with no specific recommendation on the timing for vasopressor therapy^38^. However, our experiment reflects the common clinical practice which is still today to increase blood pressure above 60–65 mmHg in critically ill patients; a survey from 2010 showed that > 87% of all responding critical care physicians would have used norepinephrine to increase blood pressure “almost always” or “mostly” in a patient with severe sepsis, even without knowing lactate concentration^59^. It could be argued that extrapolating a target blood pressure used clinically without a solid scientific foundation to an animal model exposed to vasodilatory drugs, i.e., dobutamine, as needed based on our research protocol, makes our findings less translationally relevant. However, none of the septic animals received dobutamine, since their mixed venous oxygen saturations were always above the threshold for the use of this drug. The achievement of blood pressure targets does not necessarily guarantee a sufficient organ perfusion, which is the main goal for sepsis resuscitation. For this reason, we think that blood pressure targets could be redefined, depending on negative autonomic and vascular effects evolving with vasopressor use.

An opportunity could have been to test alternative approaches to guide hemodynamic management in this experimental setting, for example, by using measurements based on organ perfusion. However, the original protocol has been designed to particularly evaluate the contribution of specific nursing manoeuvres on organ dysfunction during sepsis resuscitation^24,60^. The proposed study analyzed the experimental data retrospectively, without the possibility to do any active interventions.

The septic animals were treated only with antibiotics for the peritonitis, and this may not reflect clinical practice in which surgical interventions are considered to eradicate the source of infection. However, in our setting, there was no bowel leak which would have allow sustained leakage of feces into the peritoneal cavity. We chose our experimental setting because we demonstrated earlier that this model mimics sepsis-induced organ failure and allows to study the pathophysiology and effects of treatment over several days^61^.

## Conclusion

This work confirmed evidence from previous studies showing that sepsis induces severe vascular and autonomic alterations, as demonstrated by reduced carotid compliance, systemic vascular resistance and arterial time constant τ, increased arterial load, and depressed arterial baroreflex sensitivity. Despite hemodynamic stabilization, reduced inflammation, and achievement of the recommended hemodynamic targets, such as, e.g., MAP > 65 mmHg, the observed alterations persisted even after 3 days of successful therapy, potentially explaining the next development of sepsis-related cardiovascular disease. In particular, increased concentrations of circulating cardiac troponin I, enduring even after days of successful therapy, may be a useful marker for stratification of patients at higher risk to develop long-term cardiac complications.

Although the proposed indices cannot explain the source of the observed cardiovascular derangements, they are able to capture the macroscopic phenotype of vascular and autonomic dysfunctions, therefore, they may be used as markers of the global cardiovascular response to sepsis and the administered/recommended treatment. In the future, they may be used to guide the vasopressor dosage in order to balance the beneficial and harmful effects of such treatment. For example, they may be exploited in the setting of adrenergic stress monitoring: if increasing dosage of adrenergic vasopressor does not result in adequate improvements of blood pressure but exacerbates autonomic and vascular dysfunction, it may be read as a signal of harm for the patient and dosing tapering or a multimodal vasopressor strategy could be considered. There is a urgent need for a new tailor, multimodal vasopressor therapy strategy in septic shock patients^62^, and these indices may be additional markers to help clinical decision making in this setting. The proposed indices are based on routinely recorded waveforms so they can be computed without additional setup, this makes them ideal candidates for a real-time bedside monitoring of cardiovascular alterations.

Moreover, the proposed indices can better detail the patient’s response to treatment, and they could be useful to evaluate the effectiveness of different therapeutic options at the cardiovascular level, overcoming the limitations of using global hemodynamic end-points. The evaluation of different treatments for septic shock resuscitation (e.g. β-blockers, central α2 agonists or vagal stimulation), could take advantage of these indices which may contribute in identifying possible cluster of patients who will benefit from these therapies and in clarifying possible mechanisms at the origin of a hopefully better outcome.

## Supplementary Information

Supplementary Information.

## Data Availability

The datasets used and/or analysed during the current study are available from the corresponding author on reasonable request.
