# Supplementary material for: Autonomic and circulatory alterations persist despite adequate resuscitation in a 5-day sepsis swine experiment
Source: Sci Rep. 2022 Nov 11;12:19279. doi: 10.1038/s41598-022-23516-y (PMC9652343; doi:10.1038/s41598-022-23516-y)
Supplement: Supplementary file 1 — Supplementary Information. [file 41598_2022_23516_MOESM1_ESM.pdf]

SUPPLEMENTARY INFORMATION

Autonomic and circulatory alterations persist despite adequate resuscitation in a 5-day sepsis swine experiment

Marta Carrara[1]*, Pietro Antenucci[1], Shengchen Liu[2], Andreas Kohler[3], Rupert Langer[4,5], Stephan M. Jakob[2], Manuela Ferrario[1]

[1]Department of Electronics, Information and Bioengineering, Politecnico di Milano, Milan, Italy

[2]Department of Intensive Care Medicine, Bern University Hospital, University of Bern, Bern, Switzerland

[3]Department of Visceral Surgery and Medicine, Inselspital, Bern University Hospital, University of Bern, Bern, Switzerland

[4]Institute of Pathology, University of Bern, Bern, Switzerland.

[5]Present address: Institute of Clinical Pathology and Molecular Pathology, Kepler University Hospital and Johannes Kepler University, Linz, Austria.

*corresponding author

E-mail: marta.carrara@polimi.it

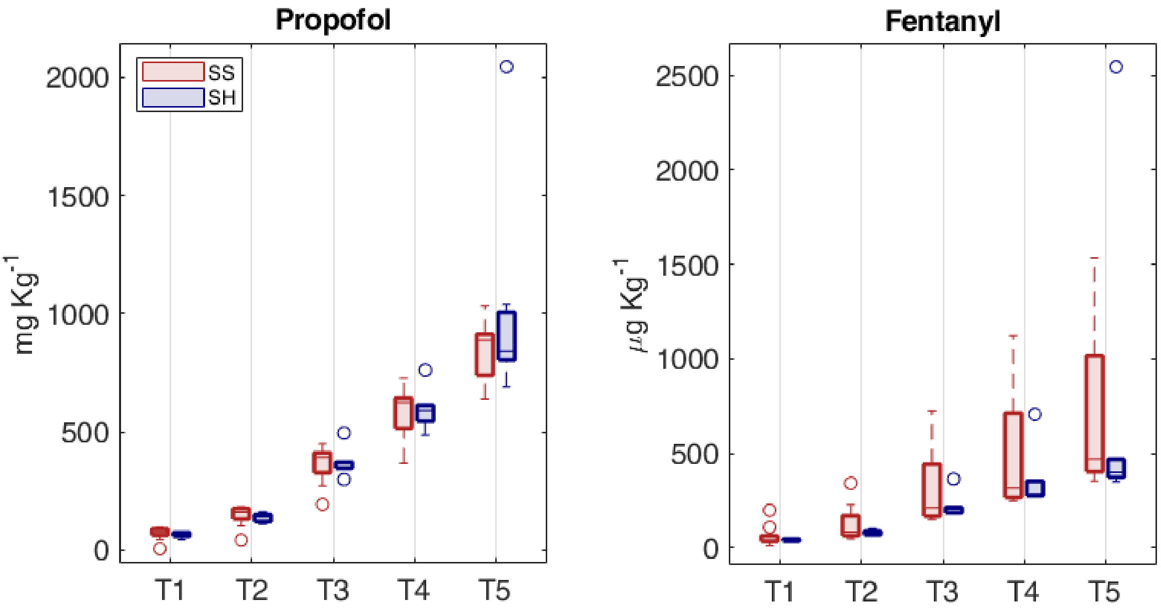

Figure S1. Distributions of administered sedative drugs for the two groups at each time point. The dosages are computed as cumulative values from the start of the experiment until each time point and are normalized with respect to each pig's weight.
